# A novel tetrahydrotetramethylbenzophenanthridinone derivative inhibits ATL cell proliferation

**DOI:** 10.1186/1742-4690-11-S1-P82

**Published:** 2014-01-07

**Authors:** Masaaki Toyama, Jun Takiguchi, Hiroshi Aoyama, Koji Yoshimura, Masaharu Nakamura, Yuichi Hashimoto, Masanori Baba

**Affiliations:** 1 Center for chronic viral diseases, Kagoshima University, Kagoshima, Japan; 2 Institute of Molecular and cellular Biosciences, The University of Tokyo, Tokyo, Japan

## 

We have previously reported that the tetrahydrotetramethylnaphthalene (TMN) derivative TMNAA is a selective inhibitor of Adult T-cell Leukemia (ATL) and HTLV-1-infected cell lines but not non-ATL cell lines and peripheral blood mononuclear cells (PBMCs). In this study, several derivatives of TMNAA were synthesized and examined for their inhibitory effect on the proliferation of various T cell lines and PBMCs by a tetrazolium dye method. The modes of cell death and cell cycle arrest were also examined by flow cytometry. Among the compounds, MN417 having a tetrahydrotetramethylbenzophenanthridinone structure was found to be a more potent inhibitor of ATL and HTLV-1-infected cell lines than TMNAA. The 50% inhibitory concentrations (IC50s) of MN417 and TMNAA for ATL and HTLV-1-infected cell lines, including S1T and MT-2 cells, were 0.59-6.6 and 2.2-34.2 μM, respectively, depending on the test cell lines. In contrast, the IC50s of MN417 and TMNAA for non-ATL cell lines, such as MOLT-4 and CEM cells, were > 20 and > 100 µM, respectively. The elimination of the TMN moiety from MN417 failed to inhibit the proliferation of ATL and HTLV-1-infected cell lines, suggesting that the TMN moiety was required for the selective inhibition of these cell lines. Furthermore, both MN417 and TMNAA hardly induced apoptosis and cell cycle arrest. Thus, although the target molecule for inhibition of ATL cells remains to be elucidated, the compounds may have potential as novel and selective chemotherapeutic agents ATL.

